# Strong Protest by the Royal British Nurses' Association

**Published:** 1916-12-09

**Authors:** W. Bezly Thorne, Comyns Berkeley, Constance Campbell Thomson, Herbert J. Paterson

**Affiliations:** Vice-Chairman and Chairman of Committees. 10a Orchard Street, Portman Square, London, W.; Honorary Treasurer. 10a Orchard Street, Portman Square, London, W.; Nurse Honorary Secretary. 10a Orchard Street, Portman Square, London, W.; Medical Honorary Secretary. 10a Orchard Street, Portman Square, London, W.


### STRONG PROTEST BY THE ROYAL BRITISH NURSES' ASSOCIATION.

*To the Editor of* THE HOSPITAL.

SIR,—The Royal British Nurses' Association desires to state publicly that it is not in agreement with several of the statements published in the communication of the Central Committee for the State Registration of Trained Nurses concerning the negotiations between that Committee and the College of Nursing, Ltd. As the Royal British Nurses' Association is one of the constituent Societies of the Central Committee, it deems it necessary to make its protest public.

At a meeting of the Executive Committee of the Central Committee, called to consider the communication referred to, the representatives of the Royal British Nurses' Association protested against its being sent to the Press, on the ground that it is inexpedient that negotiations be broken off between the Central Committee and the College of Nursing, Ltd., and that it is contrary to the best interests of trained nurses that the communication should be published.—We are, Sir, yours very truly,

(Signed)

W. BEZLY THORNE, M.D., Vice-Chairman and Chairman of Committees.

COMYNS BERKELEY, M.C. (Cantab.), Honorary Treasurer.

CONSTANCE CAMPBELL THOMSON, Nurse Honorary Secretary.

HERBERT J. PATERSON, M.C. (Cantab.), Medical Honorary Secretary.

10A Orchard Street, Portman Square, London, W., November 27, 1916.

